# Therapeutical and Pharmaceutical Notes

**Published:** 1901-03

**Authors:** W. J. Martin

**Affiliations:** Kankakee, Ill.


### THERAPEUTICAL AND PHARMACEUTICAL NOTES.

UNDER THE DIRECTION OF W. J. MARTIN, M.D.C.,
KANKAKEE, ILL.

UNCERTAINTY SURROUNDING THE ACTIONS AND USES OF MANY OF THE ALKALOIDS. Many alkaloids are agents replacing the drugs from which they are obtained. In some cases artificial alkaloids are competing with the alkaloids made in nature's laboratory, but the study of alkaloids is yet in its infancy. References to the indexes of a few volumes of the American Pharmaceutical Association will show that the nature of many of our alkaloids is as yet but little understood. Chemical investigation is adding to our positive knowledge of alkaloids and at the same time causing confusion and discrepancies in the statements made by current text-books and reference-books. We find such standards as Wood's *Therapeutics*, Maisch's *Organic Materia Medica*, and Wall's *Pharmacognosy Notes* varying in their statements as to the alkaloidal constituents of plants. This is due to the unsettled condition of affairs and depends upon the varying degree of importance which different authorities attach to current reports along the line of alkaloidal investigations. Much work was done during the sixth and seventh decade of the past nineteenth century. Still more work has been required since that time to verify or undo the records made during that period.

Alkaloids are sometimes named after one plant and then found to be of more importance as constituents of quite different drugs. Text-books on therapeutics and many of those on materia medica have little or nothing to say about berberis vulgaris, but give prominence to berberine as a constituent of hydrastis and calumba. The student also learns that hydrastin and hydrastine are quite different commercial articles. The pharmacist finds on the market an article labelled hydrastine, which is a golden-yellow, while his reference-books tell him that hydrastine is a white alkaloid ; but system is gradually replacing chaos, and before many decades of the new century pass we will have a knowledge of alkaloids which will be less confusing to the student, the pharmacist, and the practitioner.

This uncertainty as to the identity and composition of alkaloids has correspondingly perplexed physiologists and therapeutists.

For a number of years it has been positively asserted and unhesitatingly taught that calabarine of calabar bean is the physiological antagonist of physostigmine—both alkaloids of that drug. This assertion has been handed down to the 1900 editions of some works, although it is now an established fact that no such alkaloid as calabarine exists, at least in the calabar bean.

Jaborandi is another drug which has been pointed out with a considerable degree of satisfaction by teachers who enjoy dwelling upon the wise provisions of nature. They stated that jaborandi contained two alkaloids—pilocarpine being one, which associated with an antagonistic alkaloid, jaborine. Some months ago it was shown that jaborine belonged to the class of fanciful alkaloids, the commercial article being a mixture of extractive matter and other alkaloids of the plant.

At a recent meeting of the British Medical Association Prof. C. R. Marshall and Dr. H. A. D. Jewett attacked the theory of association of antagonistic alkaloids from the physiological standpoint. They joined hands with the chemists in showing that this theory is a beautiful delusion. By experiments upon lower animals and upon themselves they demonstrated that the entire therapeutic value of jaborandi is due to the pilocarpine or isopilocarpine which the leaves contain. They state positively that in no case is the inactivity of jaborandi preparations due to the presence of an alkaloid antagonistic to pilocarpine. Thus a scientific theory which always sounded like the story of William Tell is relegated to the records of the century just passed.—*Myers Bros. Druggist.*

TUBERCULOSIS AMONG CATTLE IN INDIA. In the *Annual Report of the Imperial Bacteriologist*, of India, Dr. Lingard reports as follows regarding tuberculosis: "As is known, tubercule in the human subject in India is of frequent occurrence in some localities, but up to the present I have not met with any veterinary officer who has ever observed tuberculosis in cattle in this country." Among the horses and mules at the important reserve remount depots, Hapur and Karnal, a disease appeared during the year under notice, considered to be glanders (farcy), and of a nature so serious that 112 horses and mules at Harpur were destroyed. After a careful investigation and the use of mallein (which failed in almost all the affected cases to induce reaction) the affection was decided to be lymphangitis epizoötica, described by Rivolta in 1873, and ulcerative lymphangitis, described by Nocard in 1897. This report gives it as a virulent disease, inoculable especially in equines, characterized by suppuration of the superficial lying lymphatics, and due to the presence in the tissues of a special organism—the cryptococcus farciminosus Rivoltæ—and is quite distinct from glanders. The disease develops from wounds after a period of incubation of about three months (may occupy four weeks to six months; average about three months). We are promised some further hints of the investigations into the nature and causes of disease of equines, such as surra, rookkee disease, etc.—*Veterinary Journal.*

TUBERCULAR ARTHRITIS. G. Craik, M.R.C.V.S. (*Veterinary Journal*), reports having called to see a recently calved cow, newly purchased, about six years old. Lame in off hind fetlock; great difficulty in placing weight on it, and much swelling present. Advised laxative diet, to be placed in a comfortable box, and fomentations applied thrice a day, followed by ammonia and camphor liniment. Saw it again in a week; no improvement; applied a plaster charge. In four or five days lameness greatly diminished; animal able to walk about freely, although slightly lame. On my visit on this occasion I was applying the annual tuberculin test to owner's cows, who has each animal tested once a year. This animal, having then a good appetite and a normal temperature, was subjected to the test. Reaction took place, the temperature rising 4° F.; breathing greatly accelerated and the animal off her food. The cow was destroyed and a diligent search made for tubercle. Lungs emphysematous, but no tubercle could be found; liver healthy; in fact, all the other organs normal with the exception of a few enlarged mesenteric lymphatic glands, and one caseous, in fact, nearly calcareous. At the seat of lameness, however, the tarsal bone was enlarged, red, and inflamed at the distal extremity, and on cutting through the skin on the inside of the fetlock, just where the flexor tendons go through the groove, a beautiful, soft tuberculous nodule was found.

SUTURE OF ARTERIES. This new surgical procedure of vast importance can be applied only when strict asepsis is possible and should not be tried where suppuration cannot be avoided. Its field of usefulness is in all cases of injury to a vessel where a ligature would inevitably bring about serious nutritional changes in the organ supplied by such artery, such as wounds of large arteries from puncture, gunshot, lacerations, and also in aneurisms. The finest silk and a round curved intestinal needle are used, and absolute coaptation of the edges must be produced. Overdistention as well as the slightest bruising of the vessel by instrumental pressure must be avoided.—*Wisconsin Medical Record.*

